# Reproductive health of Syrian refugee women in Lebanon: a descriptive analysis of the Sijilli electronic health records database

**DOI:** 10.1186/s12905-023-02231-4

**Published:** 2023-02-23

**Authors:** Natally AlArab, Dana Nabulsi, Nour El Arnaout, Hani Dimassi, Ranime Harb, Julien Lahoud, Lara Nahouli, Abdulghani Abou Koura, Ghaidaa El Saddik, Shadi Saleh

**Affiliations:** 1 Global Health Institute, American University of Beirut, Beirut, Lebanon; 2 School of Pharmacy, Lebanese American University, Beirut, Lebanon; 3 Department of Obstetrics and Gynecology, American University of Beirut Medical Center, Beirut, Lebanon; 4 Department of Surgery, American University of Beirut Medical Center, Beirut, Lebanon; 5 Beirut Arab University, Beirut, Lebanon; 6 Department of Health Management and Policy, Faculty of Health Sciences, American University of Beirut, Riad El Solh, Beirut, 1107 2020 Lebanon

**Keywords:** Refugees, Migration, Syrian refugees, Lebanon, Women’s health, Reproductive health

## Abstract

**Background:**

The Syrian conflict has been responsible for the highest exodus of refugees, with Lebanon hosting the greatest number of refugees per capita, which placed a significant strain on an already overburdened healthcare system. Women are the most vulnerable group in times of conflict and displacement, with sexual and reproductive health and rights often neglected. This study focuses on the obstetric characteristics and pregnancy outcomes of Syrian Refugee (SR) women in Lebanon, in Comparison to their pre-displacement data.

**Methods:**

This study is a secondary analysis of de-identified data from the Sijilli database. The data reported and analyzed were the refugees’ socio-demographics, obstetric history, pregnancy outcomes, experienced maternal and neonatal complications, breastfeeding history and duration, and contraception use and types. Data were reported in both frequencies and means/medians. Chi-square test, t-test, and ANOVA tests were used to compare pregnancies in Syria to those that happened in Lebanon.

**Results:**

A total of 1065 female records were included in this study, with 634 ever-pregnant women and the total number of pregnancies being 3272. SR women were shown to get pregnant in Lebanon at a younger age compared to cases in Syria. The number of gravidities is equal in women who got pregnant in Syria and those who moved later to Lebanon. The mean spacing between pregnancies has decreased comparing SR women who got pregnant in Syria only versus those who got pregnant in Lebanon only. Among the mixed group, the mean spacing between pregnancies as well as the prevalence of spontaneous abortions significantly increased after displacing to Lebanon. C-section rate was higher among SR women after moving to Lebanon. Also, maternal complications and not breastfeeding have increased after moving to Lebanon. A prior pregnancy was significantly associated with higher contraception use rate. The most common methods of contraception were oral contraceptive pills and intra-uterine devices.

**Conclusion:**

The C-section deliveries, spontaneous abortions and maternal complications have all increased among SR women after being displaced to Lebanon. While the age at first pregnancy, mean spacing between their pregnancies and breastfeeding rates have decreased after moving to Lebanon. SR women are less likely to use contraceptives after their displacement. It is necessary to address access to reproductive healthcare and antenatal care delivery among displaced refugee women living in informal tented settlements.

## Background

According to the United Nations High Commissioner for Refugees (UNHCR), there are approximately 27.1 million refugees in the world as of mid-2021 of which 83% of them are hosted by low-middle income countries [1]. Since its beginning in 2011, the Syrian conflict has resulted in 6.6 million refugees, representing the highest number of refugees worldwide [1, 2] with the majority having been displaced to Turkey, Lebanon, and Jordan [3]. More than ten years into the Syrian crisis, Lebanon hosts the largest number of refugees per capita, where Syrian refugees (SR) are estimated to make up 1.5 million of the country’s total population of 6.9 million [4, 5].

The 1951 Convention relating to the Status of Refugees and the 1967 Protocol relating to the Status of Refugees (the 1951 Refugee Convention) are the two primary international legal instruments that provide for the protection of the world’s refugees [6]. Lebanon is not a signatory to the 1951 Convention on the Status of Refugees, but rather, Syrian refugees in Lebanon are labeled as “displaced” and no formal refugee camps were established [6]. It is worth mentioning that the Lebanese healthcare system is privatized and fragmented, and already overburdened before the Syrian crisis, which was further aggravated by an influx of refugee populations in underserved areas [7]. Refugees face multiple barriers in accessing healthcare services which makes refugees significantly more vulnerable, particularly since they are suffering from a higher burden of disease because of displacement [8]. There has been a recent focus on the chronic conditions among SR that require greater follow-up and service provision [7]. Yet, other main topics such as women’s health were found to be the main healthcare needs of Syrian refugees in Lebanon [9].

Being a particularly vulnerable group, women were prone to lack of access to services. Moreover, the collapse of social structures has led to poor sexual and reproductive health outcomes [10, 11]. Several studies were conducted on the sexual and reproductive health (SRH) needs of Syrian refugees in the middle-east and north-africa (MENA) region. In Turkey, SR women reported early marriage, low contraception use, sexual violence, and gender-based violence [12]. In Lebanon and Iraq, Syrian refugee women were found to suffer from unmet family planning needs, in addition to high C-section rates [13]. Previous studies reported that there is at least one pregnant woman in 20–40% of Syrian refugee households [14]. Moreover, 57% of UNHCR referrals and hospitalizations in 2016 were related to pregnancy [15]. Unfortunately, the Lebanese healthcare system is unable to meet such a high demand for services. Despite partial coverage for deliveries and antenatal care, women experience multiple barriers to realized access. Based on a UNHCR survey in 2018, only 51% of women who delivered accessed the recommended 4 antenatal care (ANC) visits during their pregnancy, with 28% receiving no antenatal care (ANC) services [15]. Consequently, high rates of pregnancy and delivery complications among SR women in Lebanon were reported, attributed to poor ANC compliance [11]. Furthermore, higher rate of C-section rates among SR women in Lebanon is also reported to reach higher than 15% of the WHO recommendations, with rates up to 35% [16].

Despite the multitude of studies conducted in Lebanon on the reproductive health of SR women, few studies provide a comparison of how post-displacement figures differ from those before the beginning of the Syrian conflict. Hence, the aim of his study is to report the obstetric characteristics and pregnancy outcomes of SR women in Lebanon and to try to find out if any changes in the practices and outcomes had been noticed after these women displaced to Lebanon. So first, we aim to describe the socio-demographic characteristics and obstetric history of the SR women of childbearing-age living in informal tented settlements across Lebanon. Second, we aim to compare the socio-demographic characteristics, maternal health history and pregnancy outcomes among three groups of SR women: those who got pregnant in Syria only before their displacement, those who got pregnant in Lebanon only after their displacement and those who have pregnancy history before and after their displacement to Lebanon. The third aim is to report and compare the demographic and obstetric characteristics of contraceptive users versus non users among Syrian Refugee women as well as to report the used contraceptive methods.

## Materials and methods

### Study design

This study is a secondary analysis of cross-sectional survey data utilizing the Sijilli Electronic Health Records (EHR) Database. Sijilli is a cloud-based mobile EHR system comprising de-identified self-reported essential health information of 10,082 Syrian refugees in Lebanon [17]. The Sijilli EHR was launched in 2018, through a collaboration between the Global Health Institute at the American University of Beirut and Epic Systems Corporation- the healthcare software company. Records were collected between July 2018 and January 2020 from refugees living in Informal Tented Settlements (ITS) in four governorates (Bekaa, North and South Lebanon, Beirut/Mount Lebanon) by medical professionals and stored in a de-identified and encrypted manner on the Sijilli cloud-based server [17]. Collected data was used by the UNHCR for reporting with proportionate sample sizing to the overall Syrian refugee population.

#### Study population and collected data

Data covered by Sijilli EHR Database are divided into seven sections: socio-demographic information, social and lifestyle habits, medical and surgical history, obstetrics-gynecology (OBGYN) history, current medication use, vaccination history, and mental health screening. For this study, we only included the records of SR women above or equal to 12 years of age (assuming that it is the minimal childbearing age [18] and with available OBGYN history. Analyzed variables are socio-demographics (including age, marital status, and year of displacement from Syria to Lebanon) social habits (smoking and alcohol use), obstetric history (age of women at each pregnancy (if any), year of each pregnancy (if any), gravidity, parity and abortions of each woman. Outcomes of all pregnancies: normal delivery, C-section or abortion, in addition to the type (spontaneous versus elective abortion) and method of previous abortions were studied. Complications during pregnancies as well as during and after delivery were included. Data on breastfeeding history and contraception use was also analyzed. A variable named “mean spacing between pregnancies” was also computed and analyzed.

#### Inclusion criteria and comparison groups

As aforementioned, only the data of SR women above or equal to 12 years of age and with available OBGYN history were included in the analysis. Women were divided into three main groups: (1) SR women with pregnancy history in Syria only, (2) SR women with pregnancy history in Lebanon only, and (3) women who have pregnancy history in both Syria and Lebanon. These groups were compared at the level of socio-demographics and their OBGYN history. When comparing the pregnancy outcomes and breastfeeding duration, pregnancies in Syria only were compared to pregnancies in Lebanon only while a subgroup analysis was done to compare pregnancies in Syria to those in Lebanon among the same mixed group. Only married SR women were evaluated for contraception use and the chosen method.

### Data analysis

The collected data was coded and exported to SPSS version 26. Demographic data such as age at data collection, age at pregnancy, gravidity and parity per woman, spacing between pregnancies, and breastfeeding duration were presented as mean (SD) or median (min–max range) if data was skewed. Categorical variables such as age groups, marital status, year of arrival, alcohol and smoking status, outcomes of all and the first pregnancy (including delivery type and abortion history), outcome and complications of pregnancy, and breastfeeding history were presented using frequency and percentage. Data were tested for statistical significance by Pearson’s chi-square test or t-test when comparing two groups and ANOVA Test when comparing three groups. Logistic regression analysis was performed to study the impact of high gravidity and parity on contraception use by SR women. A p-value of ≤ 0.05 was used to denote statistical significance with a 95% confidence interval.

## Results

### Socio-demographic characteristics of the Syrian refugee women

A total of 1065 SR women above or equal to 12 years of age were included in this study. The mean age of these females was 28.8 ± 13.77; the majority of which were 18 to 35 years old (40.5%). Almost all the cohort of the SR women were ever married (98.8%) and up to 60.4% of them were still married at the onset of data collection. Among these SR women, 59.5% were ever pregnant in their lifetime. The mean age of these women was 36.3 ± 11.54 years old. Among the ever-pregnant women, 40.5% were between 18 and 35 years, 2.1% were below 18 years old and the rest were above 35 years old. The minimum age of a SR woman who was ever-pregnant was 12 years. Approximately, two-thirds (67.1%) of these women arrived in Lebanon between the years 2011 and 2014. Up to 89% of the SR women are non-smokers and do not drink alcohol (99.8%). It was shown that SR women who had ever been pregnant have higher rates of positive current smoking status (14.3%) compared to the whole cohort (9.8%) (Table 1).

**Table 1:** The Socio-demographic characteristics and social habits of SR women living in Informal Tented Settlements (ITS) in Lebanon

	Females > 12 years of age (N = 1065)		Females > 12 years of age who got pregnant (N = 634)	
	N	%	N	%
*Age (Mean ± SD)*	28.8	13.77	36.3	11.54
12–17 years old	320	30.2%	13	2.1%
18–35 years old	429	40.5%	328	52.2%
> 35 years old	310	29.3%	287	45.7%
Missing	6		6	
*Marital status*				
Single	13	1.2	10	1.6%
Married	643	60.4%	595	94.0%
Divorced	4	0.4%	4	0.6%
Separated	385	36.2%	5	0.8%
Widowed	19	1.8%	19	3.0%
*Missing*	1		1	
*Year of arrival to Lebanon*				
2010 and before	44	4.1%	26	4.1%
2011 till 2014	715	67.1%	438	69.1%
2015 till 2019	306	28.7%	170	26.8%
*Tobacco use*				
Non smoker	843	88.9%	493	84.1%
Smoker	93	9.8%	84	14.3%
Ex-smoker	12	1.3%	9	1.5%
Missing	117		48	
*Alcohol use*				
No	889	99.8%	544	99.8%
Yes	2	0.2%	1	0.2%
Missing	174		89	

### Demographic and obstetric characteristics of SR women who have pregnancy history in Syria only, Lebanon only and both Syria and Lebanon

A total number of 653 pregnant women with 3272 pregnancies were reported in this study. Table 2 shows the difference of ever-pregnant women who were divided into three categories: 193 women got pregnant in Syria only, 141 women got pregnant in Lebanon only and 300 women had a history of pregnancy before and after their displacement to Lebanon. Comparison of age at first pregnancy between the three groups showed significant differences. SR women who were pregnant in Syria only were the oldest) and those who got pregnant only in Lebanon were the youngest. The majority of SR women got their first pregnancy between the age of 18 and 35. The percentage of young pregnant women was tripled among those who got pregnant in Lebanon exclusively. The highest rate of young pregnant women (below 18 years) at first pregnancy was found to be among those who got pregnant only after their displacement to Lebanon. Median gravidity of 5–6 pregnancies per woman was reported among those who got pregnant in Syria only and SR women who got pregnant before and after their displacement. Among SR women who got pregnant for the first time after their displacement, the median gravidity per woman was 2. This may correspond to the young age of women among these categories hence they are still in childbearing age and will get pregnant later.

**Table 2:** Demographic and obstetric characteristics of SR women who have pregnancy history in Syria only, Lebanon only, and Mix (both Syria and Lebanon)

	Total Pregnancies N = 634 Women Nʹ = 3272 Pregnancies	Pregnancies in Syria only N = 193 Women Nʹ = 1254 Pregnancies	Pregnancies in Lebanon only N = 141 Women Nʹ = 326 Pregnancies	Mixed Pregnancies N = 300 Women Nʹ = 1692 Pregnancies	p-value
	%	%	%	%	
**Age at data collection**					
Mean (SD)	36.3 (11.54)	47.3 (11.39)	25.0 (6.35)	34.6 (6.27)	
< 18 years	2.1%	0.0%	9.3%	0.0%	
18–35 years	52.2%	17.5%	83.6%	59.5%	
> 35 years	45.7%	82.5%	7.1%	40.5%	
*Missing*	n = 6	n = 4	n = 1	n = 1	
**Age at pregnancy**					
Mean (SD)	27.2 (6.66)	28.7 (7.39)	23.0 (5.03)	26.9 (5.95)	< .001
< 18 years	4.5%	4.2%	12.7%	3.0%	< .001
18–35 years	84.0%	77.9%	85.4%	88.2%	
> 35 years	11.5%	17.9%	1.9%	8.8%	
*Missing*	n = 31	n = 21	n = 3	n = 7	
**Age at first pregnancy**					
Mean (SD)	21.6 (4.63)	22.2 (5.41)	21.3 (4.77)	21.5 (3.98)	0.006
< 18 years	15.8%	18.5%	21.4%	11.4%	0.170
18–35 years	82.8%	78.8%	76.4%	88.3%	
> 35 years	1.4%	2.6%	2.1%	0.3%	
*Missing*	n = 6	n = 4	n = 1	n = 1	
**Median Gravidity per woman (min–Max)**	5 (1–18)	6 (1–16)	2 (1–8)	5 (2–18)	< .001
**Mean spacing between pregnancies in years**	2.33 (1.75)	2.32 (1.825)	1.76 (1.16)	2.42 (1.74)	< .001

**Fig. 1:**
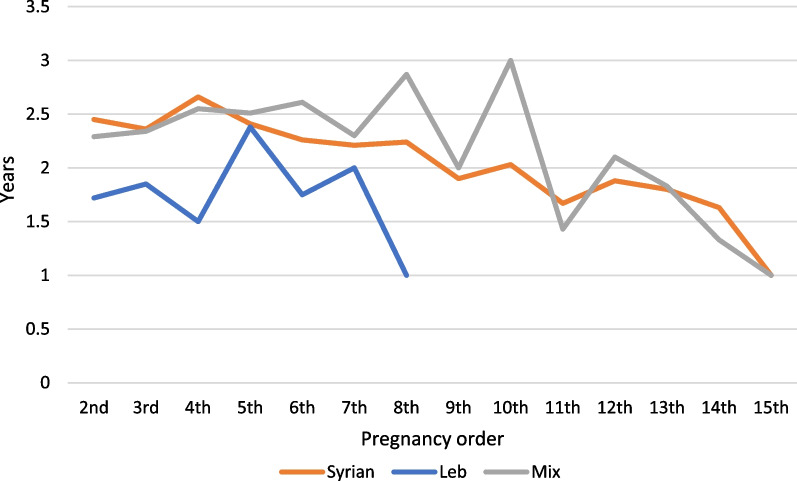
Years spacing between pregnancies by groups of SR women in Informal Tented Settlements (ITS) in Lebanon. The orange curve represents those who got pregnant in Syria only, the blue curve represents those who got pregnant in Lebanon only and the grey curve represents those who got pregnant in Syria and after they were displaced to Lebanon (Mix)

Figure 1 demonstrates the mean spacing between pregnancies (up to 15 pregnancies) among the three SR women groups. Those who got pregnant in Syria only and who got pregnant before and after displacement showed similar mean spacing especially at the first pregnancies in each group. This mean spacing of pregnancies was different in women who got pregnant only after their displacement to Lebanon which ranged between less than 6 months to almost a year. To mention that among the three groups in this cohort, it was shown that as the number of pregnancies was increasing, the spacing between them was increasing as well.

### Pregnancy outcomes of SR women who have pregnancy history in Syria only, Lebanon only, and mixed (both Syria and Lebanon): delivery method, abortions, and maternal and neonatal complications

#### Syria only versus Lebanon only cases

The rate of C-section deliveries has significantly increased to more than double between deliveries in Syria only and those in Lebanon only. Similarly, the number of overall abortion cases increased after the SR displaced and got pregnant in Lebanon for their first time. Spontaneous abortion cases were most abortions in both groups. Surgical intervention was the most reported method among SR women who had their abortions in Syria only. Pregnancy complications were significantly more common in Lebanon. Different order of complications was present among the pregnancies that happened in Lebanon only and pregnancies that happened in Syria before. Almost all complications were found to be decreased in rates in Lebanon except for post-partum hemorrhage and preterm premature rupture of membrane which showed an increase in their rates after displacement (Table 3).

**Table 3:** Pregnancy outcomes of SR women who has pregnancy history in Syria only, Lebanon only and Mixed (both Syria and Lebanon): Delivery method, abortions, and maternal and neonatal complications

	Total Pregnancies (N = 3272)	Pregnancies in Syria only (N = 1254)	Pregnancies in Lebanon only (N = 326)	p-value	Mixed Pregnancies (N = 1692)		p-value
					In Syria (N = 1085)	In Lebanon (N = 607)	
**All pregnancies**							
**Method of delivery and outcomes**				< .001			< .001
Normal vaginal	68.9%	80.0%	52.5%		71.0%	51.4%	
C-section	12.6%	6.1%	19.3%		12.2%	23.1%	
Total abortions	14.0%	11.7%	16.3%		14.0%	17.6%	
Spontaneous abortion	13.1%	11.0%	15.6%		13.4%	15.5%	
Elective abortion	0.9%	0.7%	0.6%		0.6%	2.1%	
Unknown	4.5%	2.4%	12.0%		2.9%	7.9%	
**Method of abortion**				< .001			.123
No intervention	8.7%	1.4%	30.2%		9.2%	7.5%	
Medical intervention	3.7%	2.0%	5.7%		2.6%	6.5%	
Surgical intervention	2.4%	2.7%	3.8%		0.7%	3.7%	
Unknown method	85.2%	93.9%	60.4%		87.5%	82.2%	
**Complications of pregnancy (yes)**	10.2%	8.4%	16.9%	< .001	9.8%	15.5%	< .001
Gestational hypertension	4.2%	7.5%	5.5%	.585	2.8%	2.1%	.751
Gestational Diabetes Mellitus	3.0%	2.5%	1.8%	.792	0.9%	6.4%	.053
Eclampsia/pre-eclampsia	29.0%	31.3%	14.5%	.026	37.7%	25.5%	.065
Preterm Premature Rupture of Membranes (PPROM)	6.6%	6.3%	10.9%	.331	3.8%	7.4%	.225
Infectious disease	10.4%	12.5%	10.9%	.779	7.5%	11.7%	.317
Pre-term delivery	6.3%	5.0%	1.8%	.648	8.5%	7.4%	.786
Post-partum hemorrhage	6.6%	6.3%	10.9%	.331	4.7%	6.4%	.606
Neonatal demise	8.4%	3.8%	1.8%	.645	14.2%	9.6%	.320
**History of breastfeeding (yes)**	69.7%	74.0%	60.4%	< .001	74.9%	56.7%	< .001
Missing	n = 736	n = 266	n = 95		n = 168	n = 207	
**Breastfeeding duration (months)**							
Median (min–max)	15 (1–60)	15 (1–48)	12 (1–36)	< .001	17 (1–60)	16 (1–60)	.096
Missing	n = 346	n = 142	n = 18		n = 140	n = 46	
**Outcomes for first pregnancy**	n = 634	n = 193	n = 141		n = 300	N/A	
Method of first delivery				.001			NA
Normal vaginal	74.9%	83.6%	65.8%		73.0%	N/A	
C-section	14.0%	6.3%	18.3%		17.1%	N/A	
Total abortions	11.1%	10.1%	15.8%		9.9%	N/A	
Spontaneous abortion	10.6%	9.5%	15.8%		9.2%	N/A	
Elective abortion	0.5%	0.5%	0.0%		0.7%	N/A	
Missing	n = 32	n = 4	n = 21		n = 7	N/A	
Method of first abortion*				.051			NA
No intervention	13.4%	5.3%	31.6%		6.9%	N/A	
Medical intervention	4.5%	5.3%	10.5%		0.0%	N/A	
Surgical intervention	3.0%	0.0%	5.3%		3.4%	N/A	

^*^Percent out of abortion

Among the first pregnancies in each group, normal vaginal delivery was more common among pregnancies in Syria only (Table 3). Abortions were still more common in Lebanon compared to pregnancy cases in Syria. Also, the most common method of abortion in first abortion cases was no surgical or medical interventions in Lebanon. Abortion cases only in Syria happened either without any intervention or using medical intervention methods. Breastfeeding duration was reported to be longer among births in Syria only compared to births exclusively in Lebanon.

#### Syria versus Lebanon delivery cases among mixed group

The percentage of C-sections was almost double after they displaced to Lebanon. Also, the abortion cases increased. The most common abortion method was non-medical and non-surgical both before and after displacement. Pregnancy complications were significantly increasing after reaching Lebanon. In pregnancies that happened in Syria, eclampsia/ pre-eclampsia, neonatal demise, and preterm delivery were the most common pregnancy complications. In addition to the aforementioned complications, infectious diseases were also commonly reported among the SR women who were pregnant in Lebanon only (Table 3). Breastfeeding was more common in the Syrian deliveries group with an almost equal duration of breastfeeding to those happened in Lebanon. The percentage of women who used to breastfeed in their previous pregnancies in Syria has decreased however the duration was the same.

### Contraception use

Data showed that contraceptive use was exclusive among ever-pregnant SR women. Only a quarter of the ever-pregnant SR women have reported the use of any type of contraceptive method of which none was below 18 years of age (Table 4). Contraceptive users were more likely to be those who have pregnancy histories both in Lebanon and Syria. Logistic regression analysis was made to study the gravidity and parity impact on contraception use. No significant association was documented. The most common contraceptive method was oral contraceptive pills (OCPs) followed by intrauterine Device (IUD) and condoms respectively. Other methods of contraception were reported but with lower percentages among SR women (Table 5).

**Table 4:** The demographic and obstetric history among contraceptive users of Syrian Refugee women living in Informal Tented Settlements (ITS) in Lebanon

	Women who have been pregnant at least once (N = 634)				P-value
	Ever-contraceptive users (N = 161)		Non-contraceptive users (N = 473)		
	N	%	N	%	
Age					.043
12–17 years	0	0.0%	13	2.8%	
18–35 years	92	57.9%	236	50.3%	
> 35 years	67	42.1%	220	46.9%	
*Missing*	2		4		

	Mean	SD	Mean	SD	P-value
Number of gravidities	5.29	2.729	5.12	3.256	.503
Number of parities	4.43	2.185	4.28	2.876	.479
Place of pregnancies					< .001
All Pregnancies in Syria	40	24.8%	153	32.3%	
All Pregnancies in Lebanon	23	14.3%	118	24.9%	
Mixed Pregnancies*	98	60.9%	202	42.7%	

^*^Pregnancies that happened in both Syria and Lebanon by the same SR woman

**Table 5:** The used contraceptive methods among SR women

	Total contraceptive users N = 161	
	N	%
Oral contraceptive pills (OCP)	59	36.4
Intrauterine device (IUD)	42	26.5
Condoms	20	12.3
Coitus interruptus (Withdrawal)	8	4.9
Sterilization	7	4.3
Calendar/rhythm method	3	1.9
Progesterone Implant/injection	2	1.23

## Discussion

This study examined the reproductive health status of SR women in Lebanon by utilizing the Sijilli database, with a comparison to pre-displacement data. Comparing pre- and post-displacement data provides health care providers and health policy makers with a better understanding of sexual/reproductive outcomes in a refugee population residing in the precarious living environment of refugee camps rather than solely focusing on reproductive health of resettled refugees in high-income countries. Increased risk of maternal complications, C-section deliveries and abortions as well as increased risk of adolescent pregnancy and short spacing between pregnancies of the same SR women are indicators of the difficult circumstances due to forced displacement. The latter may have physiological consequences on reproductive health, while limited access to reproductive and antenatal health care contributes to poor obstetric outcomes as clearly evident in the findings of this study.

Child pregnancy was noted among the three comparison groups with the highest percentage reaching 21.4% as first pregnancy in SR women after their displacement to Lebanon. Our results were concordant with a study conducted by the United Nations Population Fund (UNFPA) found that one-third of SR women in Lebanon between the ages of 20–24 were married before the age of 18 [19]. Similar results were reported in other studies conducted in Lebanon, Jordan and Turkey as well [20–22]. Prior to the crisis in Syria, child marriage took place among 13% of girls under the age of 18, but since then, forced displacement has led to a major increase in this number [23–25]. It is estimated that more than 500 women and adolescent girls die daily due to complications related to pregnancy and childbirth in humanitarian and emergency settings [26]. Losing support from their family and social networks, financial hardships, and disrupted education, being exposed to unsafe environment, human trafficking, risky occupations, and abuse were all found to be common motives for child marriage [26–28].

Pregnancy outcomes in this study found that 14.0% of all pregnancies ended in abortions, the overwhelming majority of which were spontaneous. This rate has significantly increased among SR women after they were displaced to Lebanon. Such results raise a huge concern on the actual cause of these abortions, which may be due to limited access to antenatal care, or limited access to contraceptives, or due to experiencing high levels of sexual violence, especially after their displacements [29]. Studies reported higher percentage of miscarriage (23%) among SR women in Turkey [30, 31]. Such discrepancy in results may be contributed to either language barriers or limited data resources which were collected via medical records and thus are likely to be underreported, as some patients could have suffered a miscarriage early on in their pregnancy without seeking medical attention. What this study adds specifically is the self-reported data, with results closer to the internationally recognized numbers. Literature revealed that women who have experienced psychological stress, and traumatic events are more likely to have a miscarriage [32, 32] putting them at an increased risk for developing mental health issues [34]. Literature on this, however, remains very limited the impact of migration on abortion rates should be further examined in detail.

Our study found that the C-section frequency among the reported deliveries in our cohort was 12.6%. Unfortunately, the C-section rates was doubled after the SR women displaced to Lebanon irrespective if it was the woman’s first pregnancy or not. Our results were in line with a studies done among the SR women in the MENA region and showed that there was an increase in C-section rates both within Syria and among refugees since the conflict began [16, 35]. Some potential explanations for this high rate are limited access to and utilization of antenatal care increasing the risk for high-risk pregnancies, predominance of male obstetric providers or UNHCR’s financial coverage of most C-sections [16]. Furthermore, communities that host displaced people may face the challenge of delivering obstetrical care without a sufficient number of trained providers available for labor management and safe deliveries, especially in rural areas [36]. Numerous studies have shown that refugee populations are more likely to suffer from adverse pregnancy outcomes [37–39], a finding well observed in our study.

Pregnancy complications were significantly more common in Lebanon as compared to pregnancy complications in Syria. Similar to literature [35], neonatal death was among the highest pregnancy adverse outcomes experienced among SR women in their pregnancies before their displacement reaching 14.2%. However, this rate decreased significantly upon their displacement to Lebanon. Globally, this percentage was found to be 2.1% [40] and in the previously mentioned study, there was no significant difference in neonatal death between refugee and non-refugee women [41]. The obtained results may be attributed to the poor maternal health access of SR women settling in ITS. Literature showed that although coverage of ANC in Lebanon remains at similar levels to pre-war Syria (up to 87.7%), whereas coverage of at least four ANC visits is much lower (in non-camp refugee populations) [35]. The increase in neonatal mortality may be a great trigger to improving timely access and equitable provision of appropriate care which should be a priority for the health system in Lebanon.

According to the United Nations Children’s Fund (UNICEF), 95% of children worldwide have been breastfed at some point in their lives [42]. Results revealed that breastfeeding was a more common practice among Syrian women before displacement. However, this practice has decreased significantly after their displacement to Lebanon among women who had previous deliveries in Syria and used to breastfeed. A study in Lebanon and Turkey showed that a much lower percentage of breastfeeding among SR women [43]. Bottle feeding has been associated with an increase in hospitalizations due to infections [44], which is especially important in refugee settings where access to clean water is not always possible [45]. A study conducted in Lebanon further elaborates on the infant and young child feeding not being given priority within the humanitarian field when it comes to supporting breastfeeding in the Syrian refugee crisis [46].

Women who had been pregnant at least once were significantly more likely to use contraception methods when compared to women who had not been pregnant. This can be explained by the recurring misconception of contraception causing infertility, thus increasing the chances of using them after having been pregnant at least once [47–51]. The mean spacing of pregnancies has been noticed to significantly decrease after displacement to Lebanon. Numerous studies have found that refugee populations have unmet contraception needs (14, 64, 65), which would explain the increase in childbearing. Among Syrian refugees specifically, a study found that contraceptives in Lebanon were unaffordable, compared to their availability for free in Syria (59). Syrian refugees were unaware of specific free services that cover sexual and reproductive health in Lebanon (66). OCPs and IUDs were the most prevalent contraception methods, which is similar to another study of the Syrian refugee population in Lebanon [51], and before the conflict [52]. According to UN estimates, although female sterilization and male condoms are the most prevalent worldwide [53], yet, OCPs (10%) and IUDs (9.5%) were the most prevalent contraceptives among women between 15 and 49 years in North Africa and Western Asia. The use of male condoms was prevalent in only 12.3% of records in our study. The low prevalence of male condom use has been documented among Syrian refugees in Lebanon [51] and its lack of use is largely associated with resistance from the men.

The strength of this study is that it is based on data from a large sample size which makes it representative of the Syrian refugee population living in informal tented settlements in Lebanon. On the other hand, the limitations of this study are the non-random sampling of the database and the recall bias arising from the self-reporting nature of the Sijilli database. Hence, a lot of missing data was encountered. No imputation of data was done but the missing data were removed from the final frequency.

## Conclusion

In this study, the reproductive health data of Syrian refugee women living in informal tented settlements in Lebanon was collected and analysed. Results showed that SR women were getting pregnant at younger age after their displacement. However, the SR women’s frequencies of C-section deliveries, spontaneous abortions, and maternal complications have all increased respectively, after being displaced to Lebanon. While the mean spacing between their pregnancy and breastfeeding rates has decreased after moving to Lebanon, SR women are less likely to use contraceptives after their displacement. It is necessary to address access to reproductive healthcare and antenatal care delivery among displaced refugee women living in informal tented settlements to control maternal health hazards among this population in Lebanon. Targeting the issue of increased childbearing of the Syrian refugee population after their displacement requires a more integrated approach, by increasing awareness on maternal health complications, providing education to dispel misconceptions, the risk of spontaneous abortion on women as well as the importance of normal delivery when it is a choice. In addition, governments and health care practitioners should increase SR women’s awareness and education about the importance of contraceptives, and breastfeeding.

## Data Availability

The datasets generated and/or analysed during the current study are not publicly available due to the confidentiality of data but are available from the corresponding author on reasonable request.

## Abbreviations

SR: Syrian refugee
UNHCR: United Nations High Commissioner for Refugees
STIs: Sexually transmitted infections
ANC: Ante natal care
WHO: World Health Organization
HER: Electronic health records
ITS: Informal tented settlements
NVD: Normal vaginal delivery
IUD: Intra uterine device
OCP: Oral contraceptive pills
UNPF: United Nations Population Fund
UNICEF: United Nations Children’s Fund
